# MicroRNAs in the Diagnosis of Digestive Diseases: A Comprehensive Review

**DOI:** 10.3390/jcm14062054

**Published:** 2025-03-18

**Authors:** Mirela Livia Popa, Cristian Ichim, Paula Anderco, Samuel Bogdan Todor, Diana Pop-Lodromanean

**Affiliations:** Faculty of Medicine, Lucian Blaga University of Sibiu, 550169 Sibiu, Romania; liviamirelapopa@yahoo.com (M.L.P.); samuelbogdant@gmail.com (S.B.T.); didi_lodro@yahoo.com (D.P.-L.)

**Keywords:** miRNA, inflammatory bowel disease, colorectal cancer, non-alcoholic fatty liver disease

## Abstract

MicroRNAs (miRNAs) have emerged as crucial regulators in digestive pathologies, including inflammatory bowel disease (miR-31, miR-155, and miR-21), colorectal cancer (miR-21, miR-598, and miR-494), and non-alcoholic fatty liver disease (miR-21, miR-192, and miR-122). Their capacity to modulate gene expression at the post-transcriptional level makes them highly promising candidates for biomarkers and therapeutic interventions. However, despite considerable progress, their clinical application remains challenging. Research has shown that miRNA expression is highly dynamic, varying across patients, disease stages, and different intestinal regions. Their dual function as both oncogenes and tumor suppressors further complicates their therapeutic use, as targeting miRNAs may yield unpredictable effects. Additionally, while miRNA-based therapies hold great potential, significant hurdles persist, including off-target effects, immune activation, and inefficiencies in delivery methods. The intricate interplay between miRNAs and gut microbiota adds another layer of complexity, influencing disease mechanisms and treatment responses. This review examined the role of miRNAs in digestive pathologies, emphasizing their diagnostic and therapeutic potential. While they offer new avenues for disease management, unresolved challenges underscore the need for further research to refine their clinical application.

## 1. Introduction

MicroRNAs (miRNAs) belong to a group of short, non-coding RNA sequences, generally spanning between 19 and 24 nucleotides in size [1,2]. These endogenous and evolutionarily conserved molecules exert their function by attaching to specific messenger RNAs, leading to translational repression and gene silencing [3,4]. MiRNAs act as essential modulators of gene expression at the post-transcriptional stage, profoundly impacting diverse cellular processes [5]. Furthermore, miRNAs are ubiquitously expressed across all eukaryotic cells [6].

These molecules are detectable in a variety of biological fluids, including urine, saliva, milk, cerebrospinal fluid, feces, and, most notably, human peripheral blood [7,8,9]. The latter is particularly significant, as circulating miRNAs exhibit remarkable stability, making them highly advantageous for diagnostic and research applications. Moreover, miRNAs are pivotal in the progression of numerous diseases, with certain ones being uniquely associated with specific pathological conditions. This underscores their promise as both biomarkers and potential therapeutic targets.

Digestive pathologies are highly heterogeneous and interact with complex pathophysiological mechanisms. The digestive system operates efficiently through the complex interplay of different cell types within the gastrointestinal tract, governed by multiple regulatory mechanisms, including translational, transcriptional, post-translational, and post-transcriptional controls [10]. As crucial regulators of gene expression, miRNAs are essential for preserving this intricate balance. Their dysregulation can profoundly disrupt normal gastrointestinal physiology, contributing to the onset and progression of a wide spectrum of digestive disorders [11]. This understanding, coupled with consistent alterations in miRNA expression profiles observed across various conditions, has spurred significant interest in their potential applications. Researchers have focused on leveraging miRNAs for early disease detection, prognostic evaluation, diagnostic classification, and predicting drug responses in a range of digestive pathologies, underscoring their value as biomarkers and therapeutic targets [12].

This review explores the significance of miRNAs in diagnosing three prominent digestive disorders: Inflammatory Bowel Disease (IBD), Non-Alcoholic Fatty Liver Disease (NAFLD), and Colorectal Cancer (CRC). In IBD, miR-16, miR-223, miR-155, and miR-21 play key roles in regulating immune responses, epithelial barrier integrity, and inflammatory cytokine production [13,14]. At the same time, in NAFLD, miR-21, miR-192, and miR-122 influence lipid metabolism, insulin sensitivity, and hepatic fibrosis [15]. Meanwhile, in CRC, miR-21, miR-598, and miR-494 modulate tumor progression, apoptosis, and cellular proliferation [16,17,18]. A comprehensive understanding of key miRNAs and their regulatory functions offers critical insights into disease mechanisms, highlighting their potential as both biomarkers and therapeutic targets. Recent clinical and experimental findings further elucidate their functional roles, emphasizing their significance in pathophysiology. Moreover, their growing applications in diagnostics and treatment strategies underscore their relevance in advancing precision medicine.

## 2. Brief History

The investigation of miRNAs in the diagnosis of digestive pathologies represents a relatively up-to-date yet rapidly advancing area of research, characterized by several pivotal milestones that will be elaborated upon in the following sections. The initial discovery of miRNAs in 1993 marked a watershed moment in molecular biology [19]. This breakthrough occurred in the nematode model organism Caenorhabditis elegans, where a genetic screen aimed at identifying defects in postembryonic development led to the identification of these small, non-coding RNA molecules [19,20]. This discovery not only revealed the existence of a novel regulatory mechanism for gene expression but also laid the groundwork for subsequent research into the diverse roles of miRNAs in health and disease [5,21]. Researchers identified miRNAs as critical regulators of gene expression, establishing a foundation for exploring their roles across diverse biological processes [3]. Following their initial discovery, the focus of miRNA research expanded into the gastrointestinal field, with significant advancements from the late 1990s to the early 2000s [3,22]. During this period, investigations sought to uncover the functional complexity of miRNAs within the gastrointestinal tract, leading to groundbreaking insights [22].

By the mid-2000s, it became evident that miRNAs were pivotal in regulating the immune response in the gastrointestinal environment, influencing key inflammatory pathways [23]. Simultaneously, they were shown to contribute to gut barrier integrity, playing an essential role in maintaining homeostasis and preventing pathological permeability [24,25]. Furthermore, miRNAs emerged as regulators of enteric neuronal development, impacting the differentiation and maturation of enteric neurons, thereby establishing the functional neural networks necessary for gastrointestinal operations [26]. Beyond these roles, miRNAs were found to modulate gastrointestinal motility, underscoring their importance in coordinating the smooth movement of the digestive system [27,28].

With these discoveries, the diagnostic potential of miRNAs began to surface and researchers increasingly investigated their relevance in digestive pathologies. The dysregulation of specific miRNAs was linked to conditions such as IBD and CRC, where unique miRNA profiles suggested their value as diagnostic biomarkers. This progress coincided with advancements in diagnostic technologies, including miRNA profiling, quantitative polymerase chain reaction, and next-generation sequencing [29,30,31]. These innovations facilitated precise and comprehensive analyses of miRNA expression patterns, enabling researchers to differentiate between health and disease states more effectively [29,30,31].

Lately, the focus has shifted toward the clinical translation of miRNA research. Significantly, research has investigated the feasibility of miRNAs as non-invasive biomarkers for early disease identification, progression tracking, and prognostic assessment in digestive pathologies. Despite promising advancements, this area remains an active field of exploration, as researchers continue to refine miRNA-based diagnostics and unravel their full therapeutic potential.

## 3. Clinical Studies and Experimental Models

The levels of miRNAs in serum exhibit remarkable stability, reproducibility, and consistency among individuals of the same species [32]. This characteristic is highly advantageous for research, as it enables investigators to identify patterns of anomalies and accurately correlate these findings with the pathology under investigation [7]. Over the years, numerous clinical studies have explored the presence and roles of miRNAs in digestive pathologies, utilizing both animal models and human subjects to uncover their diagnostic and mechanistic significance.

a. Inflammatory Bowel Disease (IBD)

IBD is a long-term, recurrent inflammatory condition that predominantly impacts young individuals and includes Ulcerative Colitis (UC) and Crohn’s Disease (CD) [33]. Its increasing worldwide prevalence presents a major public health challenge [34,35]. Currently, the gold standard for IBD diagnosis involves a combination of endoscopic evaluation and histopathological examination of biopsy specimens, allowing for precise differentiation between UC and CD while also assessing disease severity [36,37]. However, due to the invasive nature of these procedures, there is increasing interest in developing minimally or non-invasive diagnostic alternatives [38]. Among various potential biomarkers, miRNAs have gained recognition as promising tools for detecting and tracking IBD progression such as miR-16, miR-155, and miR-21 [13,39]. The rising global prevalence is concerning, as more individuals are being diagnosed with IBD [35].

Research on miRNAs in IBD began in 2008, which demonstrated that miRNAs regulate chemokine expression in colonic epithelial cells and that their expression is altered in IBD-affected tissues. Since then, significant progress has been achieved, especially in assessing miRNAs as potential biomarkers for diagnosing and predicting the progression of IBD [40]. This is attributed to the involvement of specific miRNAs in modulating key inflammatory pathways, including NF-κB and IL-6, thereby influencing inflammatory responses [40].

In the realm of IBD research, miRNAs obtained from tissue, blood circulation, and fecal samples have been explored as potential biomarkers for diagnosing the disease and predicting its progression [14]. Modern research has predominantly focused on quantifying circulating miRNA levels through advanced techniques such as microarray profiling, real-time reverse transcription PCR, and advanced genomic sequencing technologies. Furthermore, the analysis of tissue-specific miRNA expression has been extensively conducted using in situ hybridization methodologies across multiple studies [14].

Following this line of inquiry, miR-31, miR-155, and miR-21 have been consistently identified as the most extensively studied miRNAs in the context of IBD [12]. Among them, miR-21 has garnered particular attention due to its strong association with IBD, as demonstrated in multiple studies [41,42,43]. Additionally, its functional significance has been substantiated in IBD mouse models, highlighting its potential role in disease pathogenesis and progression [12].

Developing from these discoveries, a recent study demonstrated a marked rise in serum miR-146b-2-5p levels among UC and CD patients in contrast to healthy individuals [44]. More precisely, miR-146b-2-5p expression was observed to be elevated by 2.872-fold in CD patients and 2.722-fold in UC patients, demonstrating statistical significance (*p* = 0.0043) [44]. These results indicate that miR-146b-2-5p could be a promising biomarker for differentiating IBD patients from healthy individuals, shedding light on its involvement in disease pathogenesis and potential diagnostic utility [44].

Another research study revealed that miR-16 and miR-223 levels in fecal samples, but not in serum, showed a strong correlation with essential clinical inflammation markers, including C-reactive protein and calprotectin [13]. These results underscore the potential of fecal miRNA profiling as a non-invasive and clinically relevant diagnostic tool for monitoring IBD [13,45]. Given its direct reflection of intestinal inflammation, fecal miRNA analysis may serve as a more accurate alternative to blood-based biomarkers, offering enhanced specificity for disease activity assessment and progression monitoring in IBD patients [13].

Diagnosing IBD remains challenging, especially when distinguishing CD from UC, particularly in cases where inflammation is limited to the colon. Recent research has identified miR-21 as a promising biomarker for differentiating between the two conditions [41,46]. Both quantitative in situ hybridization and quantitative reverse transcription polymerase chain reaction have demonstrated significantly elevated miR-21 expression in CD compared to UC [47]. The authors propose that miR-21 is specifically associated with the immunopathological mechanisms underlying UC [47]. In situ hybridization analyses further reveal a complex expression pattern, with miR-21 staining predominantly observed in inflammatory cells within the lamina propria and within specific subsets of epithelial cells located in partially compromised crypt structures [47,48].

**Figure 1:**
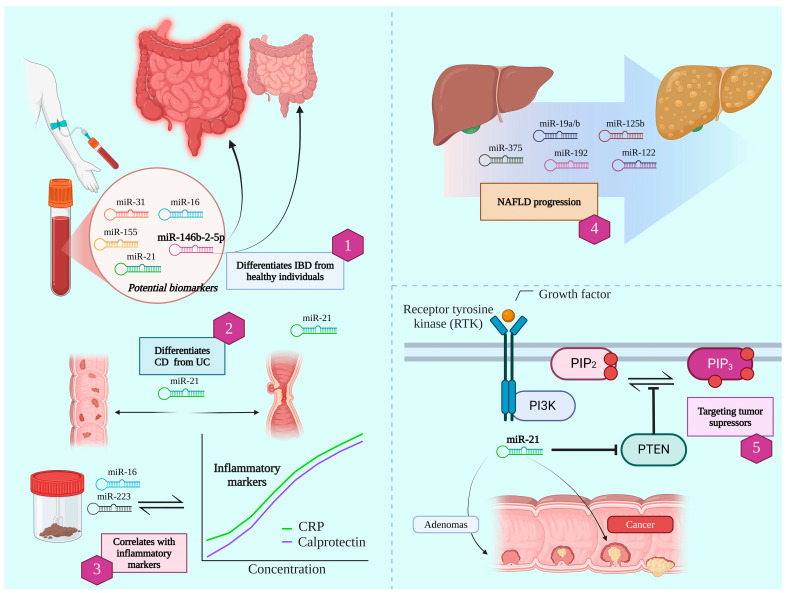
Micro-RNAs’ implications in digestive system disease: 1—Potential biomarkers like miR-146b-2-5p can differentiate between inflammatory bowel disease (IBD) and healthy individuals; 2—miR-21 can distinguish Crohn’s disease from Ulcerative Colitis; 3—Fecal miR-16 and miR-223 correlates with inflammatory markers like C-reactive protein and Calprotectin; 4—Multiple micro-RNAs can predict NAFLD progression; 5—miR-21 can target tumor suppressors like Phosphatase and Tensin Homolog (PTEN) in both adenomas and colon cancers.

A summary of the most discussed miRNAs linked to IBD in recent studies is shown in Table 1, while Figure 1 summarizes the implications of microRNAs in digestive system diseases.

b. Non-Alcoholic Fatty Liver Disease (NAFLD)

NAFLD is a disorder distinguished by an abnormal enrichment of lipids in the liver, affecting individuals with no history of alcohol consumption, viral infections, or substance abuse [51]. t is diagnosed when fat deposits exceed 5% of the liver’s total weight [52]. As the disease advances, it may develop into non-alcoholic steatohepatitis (NASH), a more severe variant characterized by steatosis, inflammation, cell necrosis, and fibrosis [53]. NAFLD is the most prevalent chronic liver disorder globally, with its occurrence and overall prevalence continuously increasing [54]. This growing burden has made it a significant global public health concern, especially because of its capacity to progress to end-stage liver failure [54]. Although the exact mechanisms driving NAFLD remain unclear, the condition is closely associated with environmental factors and metabolic dysfunctions. Growing research indicates that epigenetic regulation plays a crucial role in connecting environmental influences to the onset and progression of NAFLD [55].

NAFLD has become a major global public health issue, largely driven by its increasing prevalence and its ability to advance to end-stage liver conditions such as cirrhosis and hepatocellular carcinoma [56]. Despite extensive research, the precise pathophysiological mechanisms underlying NAFLD remain incompletely elucidated [53]. Nevertheless, it is widely recognized that environmental influences and metabolic conditions, including insulin resistance, dyslipidemia, and obesity, are key contributors to its development and progression [57].

Accumulating evidence suggests that epigenetic mechanisms serve as a critical interface between environmental influences and the onset and advancement of NAFLD [58]. These changes, such as non-coding RNAs, histone modifications, and DNA methylation, influence gene expression patterns, potentially triggering disease initiation and exacerbation [58]. MiRNAs have been recognized as critical regulators of numerous biological functions and metabolic balance, including lipid production, fatty acid and glucose breakdown, inflammation, cell proliferation, apoptosis, and necrosis [59]. Altered miRNA expression, driven by genetic, epigenetic, or environmental factors, may play a role in initiating steatosis and advancing NAFLD [59].

Several studies have documented an altered hepatic miRNA profile in both NAFLD and NASH, observed in humans and experimental models [60,61]. Differentially expressed miRNAs have also been detected in plasma samples from individuals with NAFLD. A study investigating the circulating miRNA profile in NAFLD examined 84 miRNAs, revealing distinct expression patterns linked to disease advancement [62]. The findings indicated that miR-375 miR-19a/b, miR-192, miR-122, and miR-125b were elevated in cases of simple steatosis [62]. Notably, miR-375, miR-192, and miR-122 exhibited even higher expression levels in NASH, indicating their potential as biomarkers for differentiating NASH from simple steatosis [62].

Deepening this analysis, recent exploratory studies in preclinical models have reinforced the link between circulating levels of non-coding RNAs, including miRNAs and long non-coding RNAs, and the development and progression of various liver diseases [63,64]. Notably, specific miRNAs play crucial roles in regulating complex gene networks across both acute and chronic liver conditions [65]. For instance, an analysis of diet-induced models of NASH identified elevated levels of six miRNAs: miR-505, miR-34a, miR-21, miR-192, miR-122, and miR-29a in mice with NASH. This distinct miRNA expression profile enabled accurate differentiation between NASH-afflicted and lean mice [65].

Despite the extensive body of preclinical research on miRNAs, only a few studies have advanced to clinical trials [66]. Moreover, definitive evidence regarding their diagnostic and prognostic significance in human liver diseases remains lacking [67]. Currently, biopsy remains the gold standard for diagnosing NAFLD, despite its invasive nature. Consequently, the development of biomarker-based diagnostic methods is highly valued and remains a crucial area of ongoing research.

c. Colorectal Cancer (CRC)

Colorectal cancer (CRC) is the third most prevalent malignancy worldwide, with more than 1 million new cases diagnosed each year, ranking as the second leading cause of cancer-related deaths [68]. The lack of early screening often leads to localized or distant metastases, which significantly contribute to mortality [69]. Consequently, the early detection of CRC is crucial for improving patient survival rates. In this regard, identifying new non-invasive, specific, and highly sensitive biomarkers for early CRC detection is crucial. Recent studies indicate that exosomal miRNAs, derived from liquid biopsy, show great potential as clinical biomarkers for the early diagnosis of CRC [16].

Growing research suggests that miRNAs are deeply involved in the inflammatory mechanisms of IBD and contribute to the transition from chronic inflammation to CRC development [70]. Colitis-associated colorectal cancer (CAC) is a subtype of CRC that arises in the context of IBD, potentially due to mutations in genes such as KRAS and adenomatous polyposis coli [71]. Notably, the mutation sequence in CAC often differs from sporadic CRC, with early TP53 mutations and less frequent adenomatous polyposis coli and KRAS mutations [71]. The risk of developing CAC may be further elevated in untreated IBD patients [7,71].

Multiple analyses have recognized miR-21 as among the most highly expressed miRNAs in CRC and several other malignancies. Increased miR-21 expression within the tumor stroma of CRC has been linked to reduced disease-free survival, suggesting a poorer prognosis [72]. MiR-21 directly targets tumor-suppressor genes like PTEN and PDCD4, categorizing it as an oncomiR [41]. Since miR-21 is overexpressed in IBD, it is reasonable to propose its significant involvement in the onset of CAC [41]. Supporting this theory, a study on human IBD confirmed the elevated expression of miR-21 [41].

Further exploring this evidence, current research also reported increased exosomal miR-21 expression, suggesting that miR-21 is upregulated from the early stages of adenoma development and remains elevated in advanced CRC. This indicates its potential as a diagnostic biomarker [16]. However, since miR-21 overexpression is observed across multiple cancer types, its lack of specificity for CRC may limit its effectiveness as a population-wide screening tool. MiR-155 is frequently observed to be upregulated in CRC and, akin to miR-21, serves as a biomarker indicative of unfavorable prognosis [73]. Moreover, miR-301a shows increased expression in both circulating blood and tissue samples of patients with IBD and CRC [73].

A new literature review highlighted the pivotal functional roles of miRNAs in various biological processes underlying CRC carcinogenesis [16]. Notably, miR-17-3p, miR-598, and miR-494 have been linked to the initiation and advancement of CRC. Furthermore, the upregulation of miR-1246 has been demonstrated to enhance tumor cell differentiation and invasion by suppressing cyclin G2, a crucial regulator of cell cycle progression [16]. Additionally, studies have indicated that miR-203, miR-195-5p, and miR-150-5p contribute to the dysregulation of the NF-κB signaling pathway, an essential regulator of immune response and inflammation, thereby playing a crucial role in CRC development [16].

Further expanding on miRNA regulation, it is revealed that NFIB plays a pivotal role in CRC metabolism by repressing miR-182-5p, which normally inhibits NAMPT, the rate-limiting enzyme in NAD+ salvage biosynthesis [74]. This suppression leads to increased NAMPT expression, enhancing NAD+ production, fueling cancer cell proliferation, and correlating with poor patient prognosis [74]. Meanwhile, another study identified miR-3913-5p as a tumor suppressor, revealing that its ATF2-mediated repression leads to CREB5 upregulation, which subsequently drives CRC cell proliferation, migration, and invasion [75].

Advancing this understanding, a study explored the diagnostic utility of a miRNA panel comprising the previously mentioned oncogenic miR-21 and miR-1246, along with five additional miRNAs: miR-23a, miR-223, miR-1229, miR-150, and let-7a [70]. This panel demonstrated efficacy in distinguishing patients across different stages of CRC from healthy controls. Receiver operating characteristic analysis validated the high sensitivity and specificity of this miRNA panel, underscoring its potential for clinical diagnostic applications. Furthermore, independent studies have corroborated the diagnostic relevance of miR-548c and miR-486 in CRC detection [76,77,78].

Recent studies have highlighted the critical role of microRNAs and their regulatory networks in CRC progression, tumor metabolism, and metastasis. Wang et al. demonstrated that IDH1 K224 acetylation influences the acidic tumor microenvironment by stabilizing Ago2, thereby enhancing miR-9-5p activity and downregulating NHE1, a key protein involved in extracellular acidification [79]. This process ultimately suppresses CRC cell invasion and migration. Similarly, another research study identified circTBC1D22A as a crucial regulator of autophagy, functioning as a sponge for miR-1825. By inhibiting miR-1825, circTBC1D22A restores ATG14 expression, thereby promoting autophagy and limiting CRC proliferation and metastasis [80].

Exosomal miRNAs are emerging as clinically relevant biomarkers for CRC. They enable accurate identification of early-stage disease and, according to recent studies, facilitate disease staging. This potential could significantly enhance oncological treatment by improving patient stratification and therapeutic decision-making.

## 4. Potential Therapeutic Implications

a. Inflammatory Bowel Disease (IBD)

IBD diagnosis relies on clinical manifestations, endoscopic evaluation, and histopathological examination [81]. However, these methods are not always sufficient for a definitive diagnosis. Given their role in modulating immune responses associated with IBD, miRNAs have been proposed as non-invasive and cost-effective biomarkers with potential utility in both diagnosis and therapeutic monitoring of the disease [82].

MiR-31 was recognized as a crucial factor in the transition from inflammation to neoplasia in IBD, showing significant upregulation in dysplastic and neoplastic tissues compared to non-dysplastic IBD samples [39]. This miRNA was found to disrupt essential pathways governing epithelial cell adhesion and proliferation, thereby promoting tumorigenesis [39].

A study examining the distinct miRNA expression patterns in ileal and colonic tissues of CD patients revealed notable changes in miR-192, miR-223, and miR-21 [83]. MiR-21 was associated with pro-inflammatory signaling pathways, whereas miR-223 and miR-192 were identified as key regulators of immune response and epithelial barrier maintenance [83]. The expression of specific miRNA subsets in the colonic mucosa of IBD patients was also analyzed [26]. MiR-200c, miR-150, and miR-29a exhibited significant downregulation in inactive mucosal tissues, indicating their potential role in maintaining subclinical inflammation [26]. Their normal function includes maintaining epithelial cell differentiation and suppressing inflammatory pathways, highlighting their potential role in disease modulation [26].

Drawing from these insights, another research investigated microRNA expression in the inflamed mucosal layer of the colon of individuals with active UC [84]. The authors identified the significant amplified expression of miR-155, miR-146a, and miR-124 [84]. These microRNAs were found to influence the generation of inflammation-related proteins, especially TNF-α and IL-1β, by controlling the NF-κB communication pathway in cells [84]. The study concluded that these microRNAs are key contributors to the inflammatory cascade and could be targeted for therapeutic intervention in active ulcerative colitis.

Advancing this understanding, an analysis examining the role of microRNAs in ulcerative colitis identified miR-192 and miR-375 as key regulators, with miR-192 being downregulated and miR-375 upregulated in inflamed tissues [40]. MiR-192 was shown to influence epithelial cell turnover, while miR-375 played a role in modulating macrophage inflammatory peptide-2α expression, which is essential for recruiting inflammatory cells to the gut mucosa [40].

Furthermore, a comprehensive review consolidated existing research on the involvement of microRNAs in IBD pathogenesis, emphasizing the involvement of miR-155, miR-146a, and miR-21 in immune cell differentiation, cytokine secretion, and epithelial barrier maintenance [85]. These microRNAs were proposed as promising candidates for both biomarkers and therapeutic interventions due to their broad influence on both innate and adaptive immune responses in IBD [85].

Finally, an experimental study explored the role of microRNAs in immune regulation using BALB/c mice, although specific microRNAs were not the primary focus [86]. The results revealed that microRNAs play a significant role in regulating CD4+CD25+ T regulatory cells, which are critical in maintaining immune tolerance and preventing excessive inflammation [86].

b. Non-Alcoholic Fatty Liver Disease (NAFLD)

The disease is a widespread chronic disorder influenced by a multifaceted interaction of genetic, environmental, and metabolic factors, emerging as a significant public health concern [77,87]. Current pharmacological interventions primarily aim to restore lipid homeostasis; however, their clinical utility is limited due to adverse effects such as weight gain and increased long-term mortality [88]. Consequently, these treatments are neither well-tolerated nor considered viable long-term therapeutic options for NAFLD [88].

Liver biopsy continues to be the definitive diagnostic method for NAFLD staging and the only definitive method for assessing fibrosis [89,90]. However, its invasive nature poses significant risks, including bleeding and infection [91]. Given these limitations, there is a critical need for alternative diagnostic and therapeutic approaches with reduced procedural risks. MiRNAs have emerged as promising non-invasive biomarkers with potential applications in both NAFLD diagnosis and disease management [59,92].

An investigation found elevated plasma levels of miR-122, miR-20a, miR-17, and miR-20b in individuals with type 2 diabetes mellitus and concurrent NAFLD, indicating their potential as biomarkers for early disease detection in this high-risk group [93]. Another analysis revealed that miR-24 contributes to hepatic lipid accumulation by promoting de novo lipogenesis through the activation of SREBP-1c signaling [94]. The overexpression of miR-24 facilitated SREBP-1c processing and upregulated lipogenic genes, highlighting its role in the dysregulation of lipid metabolism [94].

Building on these findings, it was reported that miR-122 is overexpressed in NAFLD-affected liver tissues and cells, whereas Sirtuin 1 expression is significantly reduced [88]. Silencing miR-122 in Huh-7 and HepG2 cells using a miR-122 inhibitor significantly reduced excessive lipid accumulation [88]. This effect was linked to the upregulation of Sirtuin 1 and the triggering of the activated protein kinase (liver kinase B1/AMP) signaling pathway, suggesting that the Sirtuin 1/liver kinase B1/AMP axis mediates microRNA-122’s role in hepatic lipid metabolism regulation [88].

Research suggests that miR-337-3p plays a protective role in NAFLD by regulating multiple metabolic pathways, reducing lipid accumulation, improving glucose homeostasis, and alleviating liver inflammation and fibrosis [95]. It also directly targets HMGCR, a key enzyme in cholesterol synthesis, linking it to cholesterol homeostasis and positioning it as a potential therapeutic target for metabolic disorders [95].

Drawing further implications from this research, the potential role of exosomal miRNA in NAFLD was highlighted [96]. MiR-199a-5p, abundantly present in fat tissue, is transported to the liver via exosomes and is potentially involved in hepatic lipid droplet accumulation [96]. It regulates MST1 levels and influences fat processing, facilitating lipid accumulation in the liver [96]. Moreover, miR-199a-5p is strongly enhanced in steatosis-prone livers of NAFLD patients and high-fat diet-induced mice [96]. These insights indicate that miR-199a-5p plays a crucial role in hepatic lipid metabolism and may serve as a promising therapeutic target for NAFLD management [96].

Another study identifies the miR-27b/MAIP1 axis as a potential therapeutic target for NAFLD, showing that miR-27b suppresses MAIP1, leading to lipid accumulation in liver cells [97]. Restoring MAIP1 expression reduced fat buildup, highlighting its role in mitochondrial lipid metabolism and offering new treatment avenues for metabolic liver diseases [97].

MiRNAs have a clear influence on controlling target genes after transcription, which are involved in the onset and advancement of NAFLD, making them highly appealing therapeutic candidates [15,98]. Modulating the expression of specific miRNAs has the potential to impact multiple clinically relevant targets, presenting promising treatment strategies [15]. Efforts are underway to standardize circulating miRNA expression profiles as non-invasive biomarkers for diagnosing NAFLD and NASH. With further validation in larger patient cohorts, these biomarkers could soon be incorporated into routine clinical practice [99].

Given that miRNAs primarily function by repressing gene expression, therapeutic strategies aim either to counteract their inhibitory effects by silencing or to restore target gene suppression by introducing sense miRNAs. Various approaches have been explored for miRNA silencing, including antisense nucleotides, miRNA sponges, competing endogenous RNAs, miRNA “erasers”, and miRNA decoys [15].

c. Colorectal Cancer (CRC)

Contemporary analyses indicate that genetic and epigenetic modifications, along with transcriptional regulation, are key contributors to the dysregulated miRNA expression observed in CRC [100]. Building on these insights, research highlights that miRNAs such as miR-296, miR-34a, miR-137, and miR-145 act as tumor suppressors by restricting the growth and spread of CRC cells [101]. In contrast, miR-21, miR-10b, and miR-552, and act as oncogenic drivers, facilitating cancer progression through the suppression of tumor suppressor genes and activation of oncogenic signaling pathways [101]. This study underscores the potential of these miRNAs as both therapeutic targets and predictive indicators for CRC diagnosis and prognosis [101].

Supporting these patterns, a systematic review assessed the effectiveness of circulating miRNAs as diagnostic biomarkers for CRC detection [102]. The analysis identified miR-21, miR-92a, and miR-29a as promising non-invasive biomarkers for CRC screening [102]. While these miRNAs demonstrate potential, the study highlighted the need for further research to establish standardized protocols and validate findings across diverse populations [102].

An examination of miR-885-3p in CRC revealed that its overexpression significantly inhibits tumor growth in HT-29 CRC xenografts in mice by modulating angiogenesis [103]. Mechanistically, miR-885-3p directly targets BMPR1A (Bone Morphogenetic Protein Receptor Type 1A), a key controller of the BMP/Smad signaling pathway, which is essential for the formation of new blood vessels [103]. By blocking BMPR1A, miR-885-3p downregulates Smad/Id1 signaling, leading to reduced endothelial cell proliferation and diminished blood vessel formation in tumors [103]. The study suggests that miR-885-3p could be a promising anti-angiogenic therapy in CRC, particularly for patients whose tumors are highly vascularized [103].

An investigation into the role of MIR22HG, a long non-coding RNA encoding miR-22, in CRC progression and response to immunotherapy revealed that MIR22HG is downregulated in CRC tissues, with its loss associated with poor patient prognosis [104]. Mechanistically, MIR22HG/miR-22 inhibits the TGFβ/SMAD signaling pathway, which is often activated in aggressive CRC and contributes to tumor progression, immune evasion, and therapy resistance [104]. The restoration of MIR22HG expression in CRC cells led to decreased proliferation, invasion, and tumor growth in vitro and in animal models [104]. Moreover, MIR22HG enhanced the response to anti-PD-1 checkpoint blockade therapy, suggesting that its restoration could improve immunotherapy outcomes in CRC patients [104].

Corroborating these insights, an analysis examined the role of miR-21 in mediating resistance to the chemotherapy drug 5-fluorouracil (5-FU) in CRC, revealing that miR-21 is overexpressed in CRC cells resistant to 5-FU [105]. Mechanistically, miR-21 directly targets and downregulates hMSH2, a crucial gene responsible for DNA mismatch repair [105]. The suppression of hMSH2 leads to decreased DNA repair capacity, contributing to chemoresistance [105]. These findings suggest that targeting miR-21 could enhance the efficacy of 5-FU chemotherapy in CRC patients [105]. Table 2 provides a summary of potential therapeutic strategies involving microRNAs in digestive pathologies.

## 5. Discussion

MiRNAs exhibit dual roles, functioning as both promoters and inhibitors of inflammation, while also acting as either cancer-driving genes or tumor suppressors, depending on the biological environment [112]. This functional versatility complicates therapeutic strategies, as modulating a specific miRNA might lead to unintended consequences [112]. For instance, inhibiting a miRNA to reduce inflammation could inadvertently promote tumorigenesis if that miRNA also functions as a tumor suppressor in certain cellular environments [112].

Despite the therapeutic potential of miRNA-based interventions, their development faces significant hurdles [113]. Difficulties in accurately identifying miRNA targets, the moderate efficacy of miRNA inhibitors, and challenges in achieving cell type-specific delivery have impeded progress [113]. Consequently, fewer than 20 miRNA-targeting molecules have entered clinical trials, with none advancing to phase III. These obstacles underscore the need for innovative approaches to harness miRNAs effectively in clinical settings [113].

MiRNA expression is highly dynamic and can be modulated by multiple factors, including interactions with the gut microbiota [114]. This reciprocal relationship complicates the development of consistent miRNA-based therapies for IBD. Alterations in gut microbiota composition can affect miRNA expression, which in turn can modulate intestinal homeostasis and inflammation, adding layers of complexity to therapeutic interventions [115].

MiRNA expression profiles can vary significantly between patients, disease stages, and even different regions of the intestine [116]. This heterogeneity poses challenges in identifying universal miRNA targets for therapeutic intervention. Personalized approaches may be required to account for individual differences in miRNA expression, complicating the development and application of miRNA-based therapies in clinical practice [117].

Modulating miRNA levels can have widespread effects due to their role in regulating multiple genes [118]. This broad impact increases the risk of adverse effects, making it crucial to develop strategies that target miRNAs with high specificity. For example, inhibiting miRNA involved in oxidative stress and inflammation could inadvertently affect other pathways, leading to unintended consequences [119]. Extending this line of thought, a comprehensive review discusses the complexities involved in miRNA biogenesis and function, emphasizing the challenges in developing miRNA-targeted therapies [120]. It highlights issues such as unintended off-target effects, difficulties in delivery mechanisms, and the intricate regulatory networks miRNAs are part of, which can complicate therapeutic interventions [120].

Other research examines advancements in delivery systems for miRNA-based therapies, noting that while progress has been made, significant obstacles remain [121,122]. Challenges include ensuring the stability of miRNA molecules in the bloodstream, achieving targeted delivery to specific tissues, and avoiding immune system activation. These hurdles must be overcome to realize the full therapeutic potential of miRNAs [121]. A previous article examined the diverse mechanisms of miRNAs in CRC, emphasizing their dual role in both promoting and suppressing tumor growth, which complicates their therapeutic use. This highlights the need for precise modulation of miRNA activity to maximize efficacy while minimizing adverse effects [123].

## 6. Conclusions

MiRNAs have emerged as promising biomarkers and therapeutic targets in digestive pathologies, including IBD, CRC, and NAFLD. Their ability to regulate multiple cellular pathways involved in inflammation, fibrosis, tumor progression, and metabolism has led to extensive research into their potential clinical applications. However, despite their promise, several controversies, ambiguities, and limitations remain, underscoring the urgent need for further investigation before miRNA-based diagnostics and therapies can be fully integrated into clinical practice.

The existing body of research on miRNAs presents both encouraging findings and significant contradictions. A major challenge lies in the dual nature of certain miRNAs, which can act as either cancer-inhibiting genes or cancer-promoting genes, depending on the specific cellular context. This complexity raises concerns about unintended consequences when modulating miRNA levels therapeutically. Additionally, miRNA expression is highly dynamic, varying based on disease stage, tissue type, and even external factors such as gut microbiota interactions. This variability complicates the standardization of miRNA-based diagnostic and treatment approaches across different patient populations.

While numerous studies have identified dysregulated miRNAs as potential biomarkers for digestive diseases, their clinical utility remains limited by several factors. The specificity and sensitivity of miRNA-based diagnostics are not yet sufficient for routine clinical application, as miRNAs often overlap in multiple disease pathways, making it difficult to pinpoint a single miRNA as a definitive diagnostic tool. Additionally, challenges in sample collection, standardization of detection methods, and variations in circulating miRNA levels further hinder their widespread adoption in clinical diagnostics.

Considering these unanswered questions, it is clear that further research is required to thoroughly clarify the precise function of small RNA molecules in digestive disorders, with a focus on:

- Standardizing miRNA detection methods to improve diagnostic accuracy and reproducibility across different laboratories.
- Clarifying the functional roles of specific miRNAs in different disease contexts to minimize unintended therapeutic effects.
- Developing targeted delivery systems that enhance the stability, specificity, and safety of miRNA-based therapies.
- Conducting large-scale clinical trials to evaluate the real-world effectiveness of miRNA-based diagnostics and treatments.

Despite the significant progress made in miRNA research, their clinical application in digestive pathologies remains in its early stages. Addressing these gaps through rigorous, well-designed studies will be crucial in determining whether miRNAs can truly revolutionize the diagnosis and treatment of digestive diseases. Until then, their role in clinical practice remains an area of exciting but cautious exploration.

## 7. Future Perspectives

Future research on miRNAs in digestive pathologies should prioritize bridging the gap between experimental findings and clinical application. Standardizing detection techniques and refining miRNA-based diagnostic models will be essential for improving accuracy and reproducibility across diverse patient populations. Additionally, elucidating the functional roles of miRNAs in specific disease contexts will help mitigate potential risks associated with therapeutic modulation. As targeted delivery strategies advance and large-scale clinical trials assess their real-world efficacy, miRNA-based approaches could transform the landscape of precision medicine in digestive diseases, offering more personalized and effective diagnostic and treatment options.

## Figures and Tables

**Table 1:** miRNA linked to IBD.

	miRNAs	Disease Subtype	Sample Type	Sample Origin	Results	Reference
1	miR-192	UC	Colonic Tissue	Human Subjects	Downregulated expression associated with inflammation severity	[40]
2	miR-375	IBD	Fecal Samples	Human Subjects	Decreased levels linked to disrupted intestinal barrier function	[49]
3	miR-484, miR-195	CD	Serum	Human Subjects	Overexpression correlated with disease activity and fibrosis	[50]
4	miR-21	UC	Colonic Tissue	Human Subjects	Upregulated expression linked to epithelial cell apoptosis	[40]
5	miR-16	CD	Fecal Samples	Human Subjects	Elevated levels correlated with disease activity	[13]
6	miR-223	UC	Fecal Samples	Human Subjects	Elevated levels correlated with disease activity	[13]
7	miR-146b-5p	CD	Serum	Human Subjects	Upregulated expression associated with disease presence	[44]
8	miR-31	UC	Colonic Tissue	Human Subjects	Upregulated expression associated with disease severity	[39]

**Table 2:** MiRNA as potential therapeutic strategies in digestive pathologies.

	mi-RNA	Digestive Pathology	Sample Source	Therapeutic Strategy	Key Findings	Reference
1	miR-21	IBD	Animal and human subjects	Inhibition of miR-21	MiR-21 is upregulated in IBD, promoting inflammation. Inhibiting miR-21 reduces inflammatory responses, suggesting therapeutic potential.	[106]
2	miR-155	CRC	Animal and human subjects	Targeting miR-155	MiR-155 is overexpressed in CRC, contributing to tumor progression. Targeting miR-155 may suppress tumor growth.	[107]
3	miR-148a	IBD	Animal subjects (mice)	Upregulation of miR-148	MiR-148 targets GP130, IL1R1, IKKα, IKKβ and TNFR2 to decrease NF-κB pathway activation, reducing inflammation in IBD.	[108]
4	miR-122	NAFDL	Animal (mice) and human subjects	Inhibition of miR-122	MiR-122 is upregulated in NAFLD and contributes to lipid accumulation in hepatocytes. Inhibiting miR-122 reduces hepatic lipid content, suggesting a therapeutic approach.	[109]
5	miR-18a	CAC	Animal and human subjects	Inhibition of miR-18a	MiR-18a is upregulated in CAC and modulates genes involved in carcinogenesis; inhibiting miR-18a may have therapeutic potential.	[107]
6	miR-222-3p	UC	Animal (mice) and human subjects	Inhibition of miR-222-3p	Blocking miR-222-3p helps relieve ulcerative colitis and colitis-related CRC by reducing oxidative damage through BRG1 regulation, which triggers the Nrf2/HO-1 cellular defense pathway.	[110]
7	miR-130a-3p	CD	Animal (mice) and human subjects	Inhibition of miR-130a-3p	MiR-130a-3p is markedly increased in CD; blocking it helps defend against colitis by regulating ATG16L1 through the NF-κB pathway, indicating its potential as a treatment target.	[111]
8	miR-34a	NAFDL	Animal (mice) and human subjects	Inhibition of miR-34a	MiR-34a is upregulated in NAFLD and promotes liver fibrosis. Inhibiting miR-34a reduces hepatic lipid accumulation and fibrosis, suggesting therapeutic potential.	[109]

## Data Availability

Not applicable.
